# Supplementary material for: Early Life Stress, Brain Development, and Obesity Risk: Is Oxytocin the Missing Link?
Source: Cells. 2022 Feb 11;11(4):623. doi: 10.3390/cells11040623 (PMC8870435; doi:10.3390/cells11040623)
Supplement: Supplementary file 1 [file cells-11-00623-s001.zip › cells-1548463-supplementary.pdf]

Material and Methods

Animals

Wild type C57BL/6 mice were purchased from Charles River Laboratories (Calco, Italy). Mice expressing GFP under the *NPY* (B6.FVB-Tg(Npy-hrGFP)1Lowl/J, stock #006417) promoter, obtained from Jackson Laboratories (Bar Harbor, ME, USA), were maintained on a C57/BL6 background. All animals were housed in plastic cages in constant environmental conditions (12 h light/dark cycle at 22◦C) with ad libitum access to food and water. Handling was limited to cage cleaning. All efforts were made to minimize animal suffering and to reduce the number of animals used. Sacrifices were carried out in accordance with Council Directive 2010/63/UE and upon approval by the Italian Health Ministry (authorization no. 405/2018-PR). All animals were 5 to 7 months old males.

Tissue processing

For morphological analyses, mice were anesthetized with 2,2,2- tribromoethanol (Avertin) (Sigma-Aldrich, Saint Louis, MO, USA) and perfused transcardially with 4% paraformaldehyde in 0.1 M phosphate buffer (PB), pH 7.4. Brains were carefully removed from the skull, postfixed in the same fixative solution for 24h at 4°C, and washed in PB. Brain regions of interest were first isolated using an adult mouse sagittal brain matrix (ASI Instruments, Warren, MI, USA). Then, free-floating 40-μm-thick coronal brainstem sections were cut with a Leica VT1200S vibratome (Leica Microsystems, Vienna, Austria) and kept in phosphate buffered saline (PBS), pH 7.4, at 4°C until use in immunohistochemical experiments. Adjacent sections were used to identify the exact location of brain nuclei of interest (brainstem, amygdala, bed nuclei of the stria terminalis, hypothalamus, and ventral tegmental area) by Nissl staining, using Paxinos and Franklin, 2001 as reference[1].

Peroxidase immunohistochemistry by light microscopy

Immunohistochemical detection of Oxytocin (Oxt) was performed on free-floating sections. First, antigen retrieval was carried out with 1% NaOH and 1% H_2O_2 (20 min), 0.3% glycine (10 min), and 0.03% sodium dodecyl sulfate (10 min). After rinsing in PBS, sections were blocked with 3% normal goat serum (in 0.2% Triton X-100; 60 min) and incubated with the Oxt primary antibody (Table 1) in PBS, overnight at 4°C. Following a thorough rinse in PBS, sections were incubated in 1:200 v/v biotinylated secondary antibody solution (in PBS; 30 min), rinsed in PBS, and incubated in avidin-biotin peroxidase complex (ABC Elite PK6100, Vector Laboratories, Burlingame, CA, USA) for 1h at room temperature. Sections were then washed several times in PBS, and finally incubated in 3,3′ diaminobenzidine tetrahydrochloride (0.05% in 0.05 M Tris with 0.03% H_2O_2; 5 min). After immunohistochemical staining, sections were mounted on slides, air-dried, dehydrated in ethanol, cleared with xylene, and covered with Eukitt mounting medium (Bio Optica Milano S.p.A, Mi, Italia). Staining was not detected when the primary antibody was omitted.

Double and triple-labelling by confocal microscopy

For double and triple-labelling staining, antigen retrieval was performed as described above. Sections were then incubated overnight at 4°C in a mixture of diluted primary antibodies (Table 1). Sections were then washed twice with PBS and incubated in a cocktail of fluorophore-linked secondary antibodies at a dilution of 1:400 in PBS for 1h at room temperature. The secondary antibodies were Alexa Fluor 488-AffiniPure F(ab')2 Fragment Donkey Anti-Guinea Pig IgG (Jackson

ImmunoResearch, Italy), Alexa Fluor R 555 donkey anti-rabbit IgG and Alexa Fluor 546 anti-sheep IgG (both from Invitrogen, Carlsbad, CA, USA). Sections were then washed twice with PBS, stained with TO-PRO3, mounted on standard glass slides, air-dried, and cover-slipped using Vectashield mounting medium (Vector). Sections were viewed under a motorized Leica DM6000 microscope at different magnifications.

In GFP-expressing NPY mice, GFP-positive cells were visualized using direct fluorescence. Fluorescence was detected with a Leica TCS-SL spectral confocal microscope equipped with an Argon and He/Ne mixed gas laser. Fluorophores were excited with the 488 nm, 543 nm, and 649 nm wavelengths and imaged separately. Images (1,024 × 1,024 pixels) were obtained sequentially from two channels using a confocal pinhole of 1.1200 and stored as TIFF files. The brightness and contrast of the final images were adjusted using Photoshop 6 (Adobe Systems, Mountain View, CA, USA).

Primary Antibodies:

Table 1:

Antibodies	Host	Dilution	Source #Cat.n.
Oxytocin	R	1:16000 (IHC)	ImmunoStar, #20068
p-STAT3 (Tyr 705)	R	1:600 (IF)	Cell Signaling Technology, #9145
Tyrosine Hydroxylase	S	1:500 (IF)	Millipore, #AB1542

GP: Guinea Pig; R: Rabbit; S: Sheep; IHC: immunohistochemistry: IF: immunofluorescence.

[1] Paxinos, G., and Franklin, K. B. J. (2001). The Mouse Brain in Stereotaxic Coordinates, 2nd Edn. San Diego, CA: Academic Press.
